# Exploring the Role of D-Dimer-to-Troponin Ratio in Differentiating Between Acute Pulmonary Embolism and Non-ST Elevation Myocardial Infarction

**DOI:** 10.7759/cureus.92124

**Published:** 2025-09-12

**Authors:** Mostafa Afifi, Yousra Nasr, Mohamed Elayady, Mohamed Ebid, Ahmad Ebrahem, Ahmed Hamed, Mona Mohamed Osman Gedi, Youssef Elbassiouni, Ibtihal Khider Fagir Salih, Huda Elkatan, Nourhan Fetouh, Raafat Elberawy, Mohamed Shabana Hussein, Mohamed Gamil Mehanna, Rothana Mohammed

**Affiliations:** 1 Faculty of Medicine, Al-Azhar University, Cairo, EGY; 2 Faculty of Medicine, Ain Shams University, Cairo, EGY; 3 Faculty of Medicine, Cairo University, Cairo, EGY; 4 Faculty of Medicine, Tanta University, Tanta, EGY; 5 Faculty of Medicine, Alexandria University, Alexandria, EGY; 6 Faculty of Medicine, Jazeera University, Mogadishu, SOM; 7 Faculty of Medicine, Mansoura University, Mansoura, EGY; 8 Faculty of General Medicine, Vitebsk State Medical University, Vitebsk, BLR; 9 Faculty of Science, King Abdulaziz University, Jeddah, SAU

**Keywords:** d-dimer, d-dimer-to-troponin ratio, non-st elevation myocardial infarction (nstemi), pulmonary embolism, troponin i

## Abstract

Background

Acute pulmonary embolism (APE) and non-ST elevation myocardial infarction (NSTEMI) are two of the most common cardiovascular emergencies with overlapping clinical presentations, which frequently produce diagnostic dilemmas. Both APE and NSTEMI share elevation in D-dimer and troponin I, rendering them less specific when utilized singly.

Objective

This study aimed to investigate whether the ratio of D-dimer to troponin I could serve as a discriminative biomarker for differentiating between APE and NSTEMI.

Methods

An observational cross-sectional analysis was performed in 40 patients with confirmed APE and NSTEMI cases. Demographic factors and laboratory variables (D-dimer, troponin I, and estimated D-dimer-to-troponin ratio) were recorded. Statistical analysis was conducted with IBM SPSS Statistics for Windows, Version 26.0 (Released 2019; IBM Corp., Armonk, New York, United States). Continuous data were reported as mean±SD, and the categorical data were given as frequencies and percentages. Comparisons between groups were made using t-tests and correlation with Pearson's coefficient, and p<0.05 was considered significant.

Results

Patients' mean age was 55.9±8.8 years for APE and 52.4±6.3 years for NSTEMI. APE patients showed much higher ratios of D-dimer to troponin (42.1±34.7) than NSTEMI patients (0.36±0.49; p<0.001). Sex-related differences were not statistically significant in both groups. Troponin I was negatively correlated with the ratio in APE (r=-0.590; p<0.001), while D-dimer was significantly correlated with the ratio in NSTEMI (r=0.798; p<0.001) in correlation analysis.

Conclusion

The D-dimer-to-troponin ratio well differentiates between APE and NSTEMI, reflecting their unique pathophysiologic mechanisms. This ratio can perhaps offer clinicians an easy, inexpensive adjunct to enhance early diagnostic precision and direct proper treatment strategies.

## Introduction

Acute pulmonary embolism (APE) and non-ST elevation myocardial infarction (NSTEMI) are two of the most prevalent acute cardiovascular illnesses seen in emergency and critical care [1]. Both present with chest pain, shortness of breath, and hemodynamic compromise and therefore often create diagnostic uncertainty during the initial evaluation process [2]. The necessity of achieving a precise diagnosis is paramount, considering the distinct treatment protocols for APE and NSTEMI anticoagulation, with the cornerstone of APE being its specific regimen, whereas anti-ischemic and antithrombotic therapies are vital for NSTEMI [3].

Laboratory biomarkers play a vital role in the diagnostic assessment of both conditions. D-dimer, which is a byproduct of fibrin degradation, is frequently utilized to either confirm or exclude the diagnosis of venous thromboembolism, including APE [4]. Cardiac troponin, which is a sensitive and specific marker of myocardial damage, is the key to diagnosing acute coronary syndromes, including NSTEMI [5]. However, both biomarkers are elevated in various clinical conditions beyond their initial target disease. APE can lead to secondary myocardial injury, resulting in a rise in troponin levels, while inflammation or comorbidities may elevate D-dimer levels in patients with NSTEMI [6].

Given these overlaps, it is improbable that either biomarker can independently differentiate APE from NSTEMI. Recent studies have suggested that the D-dimer-to-troponin ratio may serve as a more effective distinguishing marker by balancing the relative influence of thrombosis against myocardial injury. An increased ratio could indicate APE, characterized by significantly heightened fibrin turnover, while a decreased ratio would be more indicative of NSTEMI, where myocardial necrosis is more prevalent [7].

Thus, investigating the D-dimer-to-troponin ratio as a diagnostic marker may offer clinicians an inexpensive, quick, and easy adjunct to routine diagnostic regimens. This study seeks to examine whether this ratio can differentially diagnose patients with established APE from patients with NSTEMI and thereby improve early diagnostic precision and direct proper therapeutic interventions.

## Materials and methods

Study design

This was a cross-sectional, comparative, observational study with the aim of assessing the diagnostic value of the ratio of D-dimer to troponin in the differentiation between APE and NSTEMI.

Study setting

After obtaining approval from the Research Ethics Committee of Hassan Ghazzawi Hospital (approval number: DHGH-MBEC-2405), the research was carried out at Hassan Ghazzawi Hospital, Jeddah, KSA, a healthcare facility with an emergency and cardiology specialty unit, where patients presenting with acute chest pain and possible cardiovascular emergencies are regularly screened.

Study population

The study involved 80 patients who had either established APE or NSTEMI during the study period. The patients were divided into two groups: the APE group comprising patients with an established diagnosis of APE and the NSTEMI group comprising patients with an established diagnosis of NSTEMI.

Inclusion criteria

The inclusion criteria were as follows: (a) adult patients (≥18 years of age) with acute chest pain or associated symptoms; (b) patients with a confirmed diagnosis of APE according to imaging (computed tomography (CT) pulmonary angiography) or NSTEMI according to clinical presentation, electrocardiogram (ECG), and positive cardiac biomarkers; and (c) presence of laboratory measurement of D-dimer and troponin levels upon admission.

Exclusion criteria

The exclusion criteria were as follows: (a) patients with dual diagnoses of APE and NSTEMI; (b) patients with other acute or chronic illnesses that may confound biomarker levels (e.g., sepsis, disseminated malignancy, chronic kidney disease); and (c) incomplete clinical or laboratory records.

Data collection

Demographic data (age and sex) and laboratory values (levels of D-dimer in fibrinogen equivalent units (FEU/mL) and troponin I (ng/mL)) were retrieved from the medical records. For every patient, the ratio of D-dimer to troponin was obtained by dividing D-dimer by troponin I.

Statistical analysis

All the statistical analysis was conducted using IBM SPSS Statistics for Windows, Version 26.0 (Released 2019; IBM Corp., Armonk, New York, United States). Continuous variables were described in terms of mean±standard deviation (SD) and categorical variables in terms of frequencies and percentages. The normality of continuous data was checked using the Kolmogorov-Smirnov and Shapiro-Wilk tests. For between-group comparisons, the independent samples t-test was used for variables having a normal distribution, while non-parametric tests were utilized for variables that lacked normal distribution. Paired sample analysis was performed when relevant in order to assess within-group biomarker differences. Pearson's correlation coefficient was used to determine correlations between biomarkers and age, and a p-value of less than 0.05 was used as the threshold for statistical significance.

## Results

Demographic characteristics

A total of 40 patients for each group were included in the study. As shown in Table 1, the average age of APE patients was 55.9±8.8 years (range: 40-80 years), whereas NSTEMI patients averaged 52.4±6.3 years (range: 43-71 years). The distribution of sex in both groups was 26 (65%) patients were male and 14 (35%) were female. 

**Table 1:** Age and sex distribution of patients with APE and NSTEMI N: number of patients; APE: acute pulmonary embolism; NSTEMI: non-ST elevation myocardial infarction; Std. deviation: standard deviation

		N	Minimum	Maximum	Mean	Std. deviation
Age	APE	40	40.0	80.0	55.900	8.8137
	NSTEMI	40	43.0	71.0	52.417	6.3443
			Frequency		Percentage	
Sex (APE and NSTEMI)	Male		26		65	
	Female		14		35	
	Total		40		100	

Biomarker profiles

The mean D-dimer concentration in PE patients was 5.0±2.2 FEU/mL, while the mean troponin I was 0.32±0.42 ng/mL with a mean D-dimer-to-troponin ratio of 42.1±34.7. In comparison, NSTEMI patients had a mean D-dimer concentration of 0.69±0.58 FEU/mL, a mean troponin I of 7.24±12.5 ng/mL, and a significantly lower ratio of 0.36±0.49 (Table 2).

**Table 2:** Descriptive statistics of biomarkers in APE and NSTEMI patients N: number of patients; APE: acute pulmonary embolism; NSTEMI: non-ST elevation myocardial infarction; Std. deviation: standard deviation

Biomarker profile	N	Minimum	Maximum	Mean	Std. deviation
PE_D_Dimer_FEU_ml	40	1.3	10.1	5.005	2.2141
PE_Troponin_I_ng_ml	40	.03	2.00	.3210	.42645
PE_DDimer_Troponin_Ratio	40	2.000000	170.000000	42.09711668	34.666806139
NSTEMI_D_Dimer_FEU_ml	40	.1	2.6	.694	.5811
NSTEMI_Troponin_I_ng_ml	40	1.00	50.00	7.2436	12.52080
NSTEMI_DDimer_Troponin_Ratio	40	.012000	2.363636	.36294711	.499050229
Valid N (listwise)	40				

**Figure 1:**
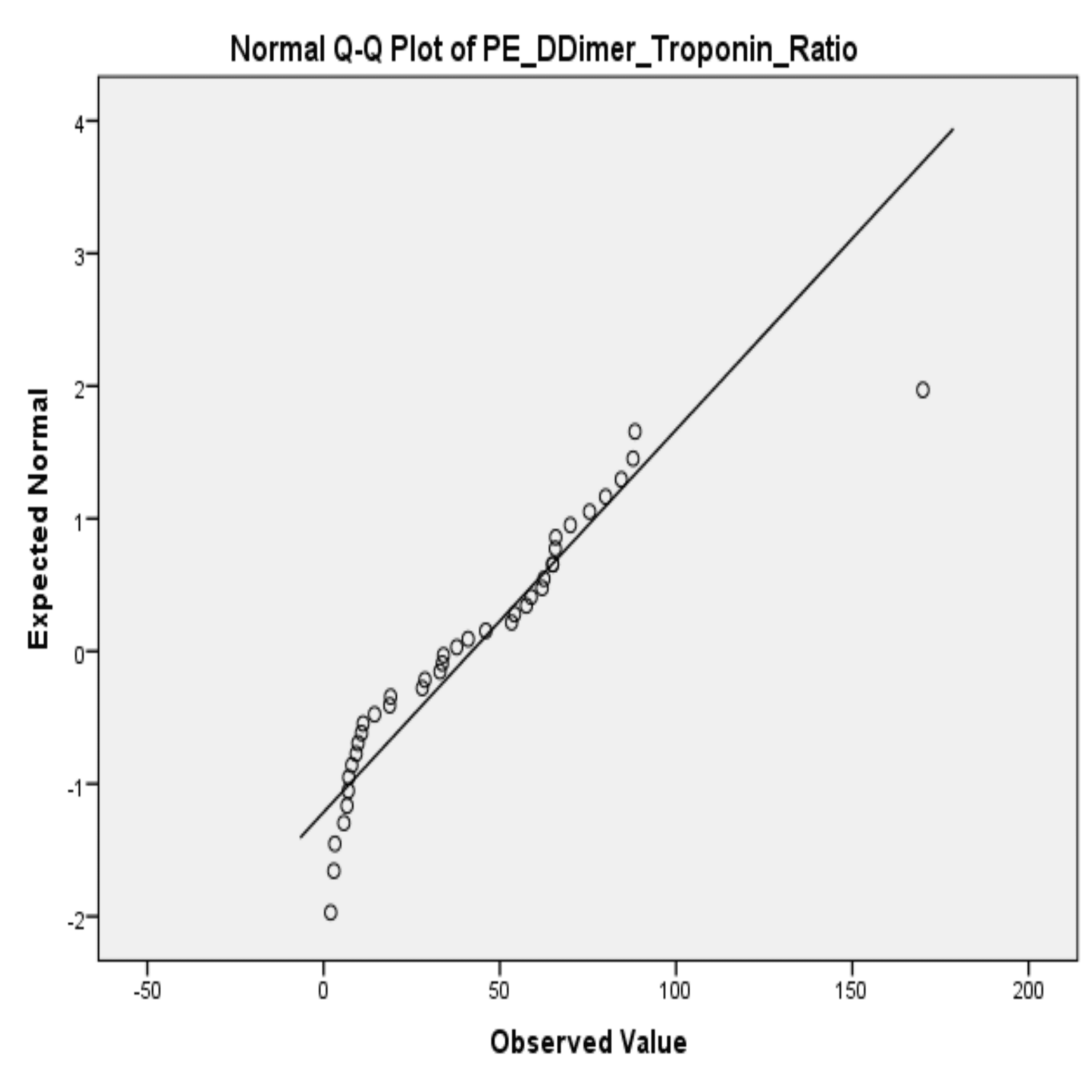
Normal Q-Q plot of D-dimer-to-troponin ratio in APE patients APE: acute pulmonary embolism

**Figure 2:**
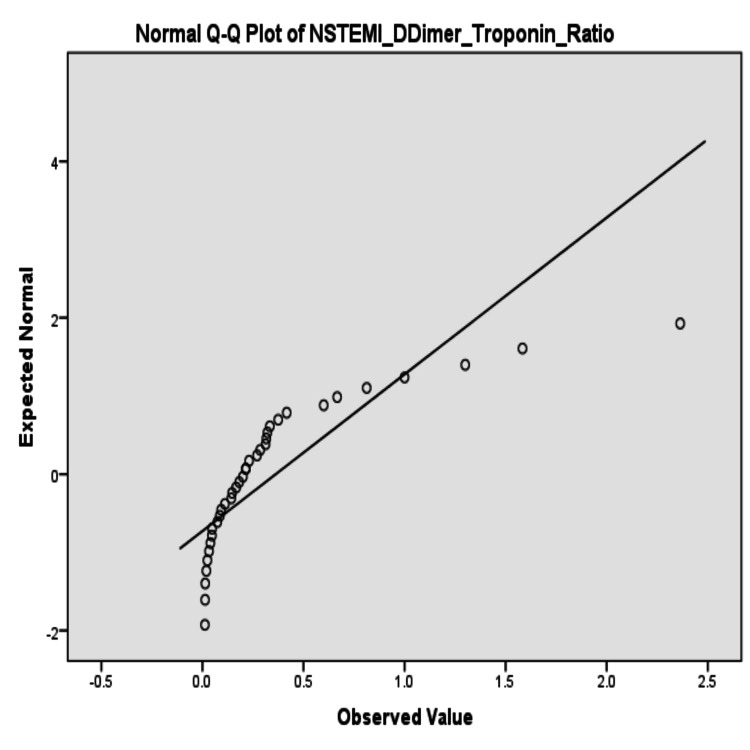
Normal Q-Q plot of D-dimer-to-troponin ratio in NSTEMI patients NSTEMI: non-ST elevation myocardial infarction

The skewness of the D-dimer-to-troponin ratios was also graphically depicted by the stem-and-leaf plots. In the APE group, the values of the ratios were right-skewed, with multiple high outliers apparent (≥170) (Figure 1, PE_DDimer_Troponin_Ratio Stem-and-Leaf Plot). For NSTEMI ratios, values were concentrated closer to the low end with fewer extreme values (Figure 2, NSTEMI_DDimer_Troponin_Ratio Distribution).

Normality analysis

Normality tests indicated that the D-dimer-to-troponin ratio for APE patients was 0.124 (p=0.125), but the Shapiro-Wilk test was significant (p=0.000) with evidence of deviation from normality. The NSTEMI ratio similarly demonstrated deviation from normality (Kolmogorov-Smirnov p=0.000; Shapiro-Wilk p=0.000) (Table 3).

**Table 3:** Tests of normality for D-dimer-to-troponin ratio in APE and NSTEMI patients ^a^Lilliefors significance correction APE: acute pulmonary embolism; NSTEMI: non-ST elevation myocardial infarction

	Kolmogorov-Smirnov^a^			Shapiro-Wilk		
	Statistic	df	Sig.	Statistic	df	Sig.
PE_DDimer_Troponin_Ratio	.124	40	.125	.877	40	.000
NSTEMI_DDimer_Troponin_Ratio	.274	40	.000	.677	40	.000

**Figure 3:**
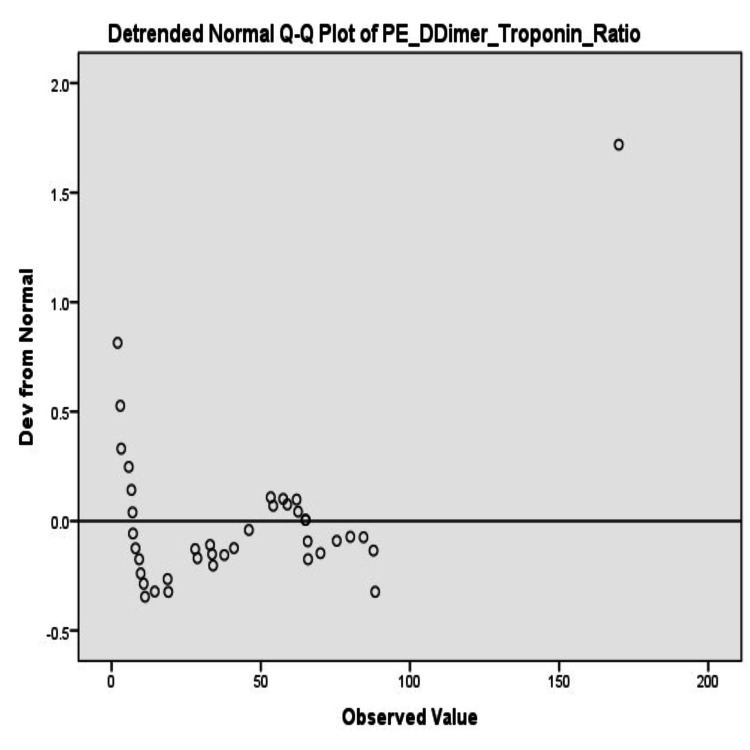
Detrended normal Q-Q plot of D-dimer-to-troponin ratio in APE patients APE: acute pulmonary embolism

**Figure 4:**
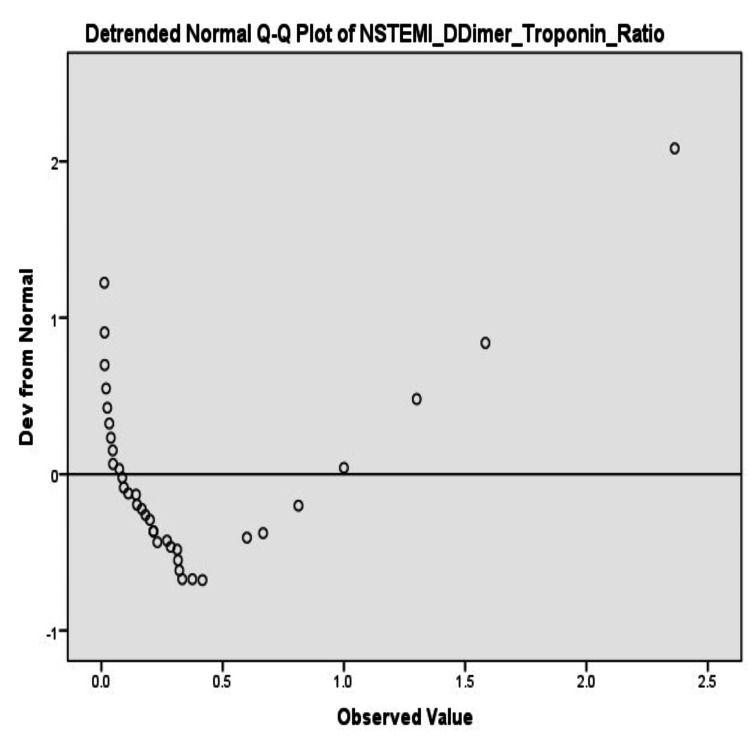
Detrended normal Q-Q plot of D-dimer-to-troponin ratio in NSTEMI patients NSTEMI: non-ST elevation myocardial infarction

The non-normal pattern of distribution was also graphically supplemented by the SPSS output frequency distribution values (Figures 3-4).

Paired samples t-test

A paired samples t-test revealed a statistically significant difference between the two groups. The mean difference in ratios was 43.8 (95% CI: 31.9-55.6), with t=7.516, df=35, and p<0.001 (Table 4).

**Table 4:** Paired samples test comparing D-dimer-to-troponin ratios between APE and NSTEMI patients APE: acute pulmonary embolism; NSTEMI: non-ST elevation myocardial infarction; Std: standard; t: t-test; df: difference

		Paired differences					t	df	Sig. (two-tailed)
		Mean	Std. deviation	Std. error mean	95% confidence interval of the difference				
					Lower	Upper			
Pair 1	PE_DDimer_Troponin_Ratio - NSTEMI_DDimer_Troponin_Ratio	43.801904750	34.968014409	5.828002402	31.970430869	55.633378631	7.516	35	.000

Correlation of the paired ratios demonstrated no significant association (r=0.093; p=0.591) (Table 5).

**Table 5:** Correlation between D-dimer-to-troponin ratios in APE and NSTEMI patients APE: acute pulmonary embolism; NSTEMI: non-ST elevation myocardial infarction

		N	Correlation	Sig.
Pair 1	PE_DDimer_Troponin_Ratio&NSTEMI_DDimer_Troponin_Ratio	40	.093	.591

## Discussion

This study examined the diagnostic value of the ratio of D-dimer to troponin in distinguishing between APE and NSTEMI, two of the most prevalent cardiovascular emergencies seen in daily practice. The presenting symptoms of both conditions are the same, including chest pain, dyspnea, and hemodynamic instability, which frequently create a clinical dilemma in the early part of the evaluation. The study aimed to evaluate whether the relative ratio between fibrinolytic products (D-dimer) and markers of myocardial damage (troponin I) was a helpful, quick, and inexpensive biomarker in the differentiation of these entities.

The findings of this study indicate that the D-dimer-to-troponin ratio gives a definite separation between APE and NSTEMI patients. The APE patients had D-dimer levels very much raised but comparatively low troponin I levels, which produced a very high ratio (mean: 42.1±34.7). The NSTEMI patients had low D-dimer levels but very high troponin I levels, which produced a very low ratio (mean: 0.36±0.49). The two groups differed significantly (p<0.001). Additionally, whereas sex differences in the APE group were noted, these were not statistically significant, and in NSTEMI patients, the ratios were almost identical in both genders. Correlation analysis also validated the ratio: in PE, troponin was inversely related to the ratio, but in NSTEMI, D-dimer was highly positively related to the ratio.

These findings confirm the initial hypothesis that the D-dimer-to-troponin ratio may serve as a discriminative biomarker, highlighting its potential to improve the diagnostic process when distinguishing between thrombotic and ischemic pathologies presenting with similar symptoms.

The diagnostic challenge of differentiating APE from NSTEMI has been thoroughly documented in previous studies. Earlier investigations have shown that both troponin and D-dimer levels are elevated in either condition, making these measurements not particularly specific when utilized independently [8]. For instance, a rise in troponin levels has been observed in as many as 50% of patients with APE, attributed to right ventricular strain and subsequent myocardial injury. Additionally, another study indicated that elevated D-dimer levels may also be present in patients experiencing myocardial infarction, possibly due to the secondary breakdown of fibrin associated with inflammation and atherothrombosis [9]. All these shared patterns make single-biomarker approaches inadequate for precise distinction.

The findings of this study support that ratio, either alone or associated with other markers, may improve diagnostic discrimination. A separate study examined the use of multi-biomarker panels for evaluating acute chest pain and proposed that the interaction between markers of myocardial necrosis and hemostatic markers offers superior discrimination compared to either parameter when considered alone [10]. The present study lends empirical basis to such an approach by showing that the D-dimer-to-troponin ratio generates a different profile for every condition: thrombosis-predominant in APE and necrosis-predominant in NSTEMI.

In addition, the large ratio difference seen in this study is consistent with results from the study that brought attention to the value of considering biomarker dynamics in differential diagnosis [11]. The ratios presented in our group are in accordance with the biological likelihood of disease pathophysiology: increased fibrinolysis in APE and dominant myocardial damage in NSTEMI.

The biologic basis of the ratio of D-dimer to troponin rests in the different pathophysiologic mechanisms of APE and NSTEMI [12]. In APE, pulmonary artery obstruction by thromboembolic material causes significant fibrin turnover, which is manifested by high levels of D-dimer [13]. Although myocardial strain can cause minor troponin release, this is typically moderate compared to coronary occlusion [14]. Therefore, the numerator (D-dimer) prevails in the ratio.

NSTEMI is facilitated by intrapartial coronary occlusion and causes myocardial necrosis [15]. It significantly increases troponin levels, with a minimum increase in D-dimer, which occurs due to either systemic inflammation or secondary prothrombotic conditions [16].** **The denominator (troponin) thus dominates and forms a low ratio. These differences in mechanisms account for the dramatic contrast noted in our findings.

The clinical value of these results is great. Diagnostic uncertainty by emergency physicians is common during the assessment of patients with acute chest pain, particularly if imaging technologies like CT pulmonary angiography or coronary angiography are not currently on hand [17]. A straightforward ratio calculated from commonly performed laboratory studies may be used as an adjunctive tool that will direct early management. For instance, an abnormally high D-dimer-to-troponin ratio would favor imaging in APE, whereas an extremely low ratio would suggest NSTEMI and warrant immediate cardiac assessment.

Additionally, this ratio can potentially decrease unnecessary testing and related expenses by more effectively guiding diagnostic workup. In settings of limited resources where advanced imaging is not readily available, the ratio might offer a useful first-level triage intervention. It also has implications for research into risk stratification since patients with borderline values might constitute overlapping pathophysiology or blended presentation.

Strengths of the study

There are a number of strengths of this study. First, it contrasts the biomarker profiles of two clinically indistinguishable yet pathophysiologically disparate conditions directly, thereby solving a frequent real-world diagnostic challenge. Second, measuring biomarkers as a ratio instead of absolute values increases discriminative capability, which is evidenced by statistically significant group differences. Third, this study employed standardized assays to measure biomarkers, guaranteeing the reliability of results. Lastly, by involving both male and female patients of varied ages, the study yields generalizable findings that can be applied to a diverse patient population.

Future directions

Following on from this work, future research would seek to confirm the D-dimer-to-troponin ratio in larger and more diverse cohorts. Multicenter prospective studies could determine standardized cutoff values for practical application. Adding other biomarkers, including B-type natriuretic peptide (BNP) or high-sensitivity troponin, may further optimize the ratio's diagnostic performance.

There is also scope for the inclusion of the ratio in machine learning-based models of prediction for acute chest pain assessment, combining biomarker information with clinical characteristics and imaging findings. These models have the potential to transform emergency diagnosis by providing swift, automated, highly accurate differential diagnoses.

## Conclusions

This research demonstrates that the D-dimer-to-troponin ratio serves as a valid biomarker for differentiating between APE and NSTEMI. Patients with APE exhibited significantly elevated ratios, reflecting the predominance of fibrin turnover, whereas NSTEMI patients showed markedly lower ratios, consistent with myocardial necrosis. These findings align with biological plausibility and existing literature, underscoring the ratio's potential as an inexpensive and user-friendly diagnostic tool. Despite certain limitations, the results provide a strong foundation for more extensive prospective studies aimed at establishing the ratio's role in standard clinical practice. The D-dimer-to-troponin ratio may improve diagnostic precision, optimize resource utilization, and facilitate the prompt initiation of effective treatment for patients experiencing acute chest pain.
